# High risk of loss to follow-up among South African children on ART during transfer, a retrospective cohort analysis with community tracing

**DOI:** 10.7448/IAS.20.1.21748

**Published:** 2017-06-28

**Authors:** Chloe A. Teasdale, Nonzwakazi Sogaula, Katharine A. Yuengling, Zachary J. Peters, Anthony Mutiti, Lungile Pepeta, Elaine J. Abrams

**Affiliations:** ^a^ ICAP, Mailman School of Public Health, Columbia University, New York, NY, USA; ^b^ Department of Epidemiology, Mailman School of Public Health, Columbia University New York, New York, NY, USA; ^c^ Port Elizabeth Hospital Complex, Department of Paediatrics, Port Elizabeth, South Africa; ^d^ Nelson Mandela Metropolitan University, Faculty of Health Sciences, Port Elizabeth, South Africa; ^e^ College of Physicians & Surgeons, Columbia University, New York, NY, USA

**Keywords:** loss to follow-up, paediatric HIV, ART, decentralization, referral, retention

## Abstract

**Introduction**: Decentralization of HIV care for children has been recommended to improve paediatric outcomes by making antiretroviral treatment (ART) more accessible. We documented outcomes of children transferred after initiating ART at a large tertiary hospital in the Eastern Cape of South Africa.

**Methods**: Electronic medical records for all children 0–15 years initiating ART at Dora Nginza Hospital (DNH) in Port Elizabeth, South Africa January 2004 to September 2015 were examined. Records for children transferred to primary and community clinics were searched at 16 health facilities to identify children with successful (at least one recorded visit) and unsuccessful transfer (no visits). We identified all children lost to follow-up (LTF) after ART initiation: those LTF at DNH (no visit >6 months), children with unsuccessful transfer, and children LTF after successful transfer (no visit >6 months). Community tracing was conducted to locate caregivers of children LTF and electronic laboratory data were searched to measure reengagement in care, including silent transfers.

**Results**: 1,582 children initiated ART at median age of 4 years [interquartile range (IQR): 1–8] and median CD4+ of 278 cells/mm^3^ [IQR: 119–526]. A total of 901 (57.0%) children were transferred, 644 (71.5%) to study facilities; 433 (67.2%) children had successful transfer and 211 (32.8%) had unsuccessful transfer. In total, 399 children were LTF: 105 (26.3%) from DNH, 211 (52.9%) through unsuccessful transfer and 83 (20.8%) following successful transfer. Community tracing was conducted for 120 (30.1%) of 399 children LTF and 66 (55.0%) caregivers were located and interviewed. Four children had died. Among 62 children still alive, 8 (12.9%) were reported to not be in care or taking ART and 18 (29.0%) were also not taking ART. Overall, 65 (16.3%) of 399 children LTF had a laboratory result within 18 months of their last visit indicating silent transfer and 112 (28.1%) had lab results from 2015 to 2016 indicating current care.

**Conclusion**: We found that only two-thirds of children on ART transferred to primary and community health clinics had successful transfer. These findings suggest that transfer is a particularly vulnerable step in the paediatric HIV care cascade.

## Introduction

UNAIDS estimated that as of 2015, 1.8 million children <15 years were living with HIV and only half (49%) were receiving antiretroviral therapy (ART) [1]. Expanding HIV care and treatment to all HIV-infected children has been challenging due to factors including testing barriers, lack of paediatric healthcare providers in many resource-limited settings (RLS), and few highly potent paediatric treatment formulations, particularly as fixed-dose combinations [2–5]. In addition, many of the first facilities offering paediatric treatment were urban tertiary centres which limited access for children in rural areas [6]. For children initiated on ART, there continue to be challenges with high rates of loss to follow-up (LTF) and mortality [7–11].

Scale up of HIV treatment in rural areas and decentralization of paediatric care to lower-level health facilities have been critical to expansion of treatment access [12,13]. A small number of studies have found that, similar to adults, children who initiate ART at tertiary health facilities and are transferred (“down referred”) to primary health clinics have comparable or better mortality and retention outcomes [14–18]. While studies have shown that LTF may be lower at primary health facilities, few analyses have examined LTF of children that occurs at the time of transfer. LTF during the transfer process may be significant; an analysis of stable adult ART patients in South Africa who were down referred found that almost 60% of all of the LTF occurred at the time of the transfer [19].

Retention of paediatric patients in HIV care is particularly important as children have more rapid disease progression and higher risk of mortality [20,21]. The high rates of LTF in children reported from many RLS are extremely concerning and more information is needed on what happens to these children. Studies of adult patients LTF have shown that 20–40% have died [22–25], 10% have “silently” transferred to other health facilities, and 6% are disengaged from care [26]. Only a few studies have described outcomes of children LTF and little is known about the reasons for disengagement from care.

South Africa’s national treatment guidelines called for decentralizing HIV care for adults and children starting in 2010 [27]. In this study, we examined children initiated on ART at a tertiary hospital in the Eastern Cape of South Africa over 11 years and documented the proportion of children with successful transfer to primary and community health clinics. We then attempted to ascertain outcomes of children LTF at the hospital, during transfer, and after successful transfer. We conducted community tracing and used the national laboratory database to find HIV-related monitoring after LTF to identify engagement in care including silent transfers and re-engagement in care after periods of non-attendance.

## Methods

We examined electronic medical records (EMR) for all children 0–15 years who initiated ART at Dora Nginza Hospital (DNH) in Port Elizabeth (Eastern Cape Province of South Africa) from 1 January 2004 to September 31, 2015. Ethical and administrative approvals were received from the Columbia University Medical Center Institutional Review Board, the University of Cape Town Human Research Ethics Committee and the Eastern Cape Department of Health.

Based on EMR data, we identified all children transferred after ART initiation to primary and community health clinics, as well as indication of care outcomes at DNH, including documented deaths. No national or provincial guidelines were available with information on clinical criteria used to select patients for transfer; however, providers at the facilities reported in discussions with the study team that they transferred patients who were stable on treatment. Children were transferred to 112 facilities; 80 in the Eastern Cape and 32 in other provinces. We examined routinely collected data from medical charts and facility registers at 16 of the health facilities in Eastern Cape; facilities were selected based on having at least 10 children transferred and were all in the area surrounding the city of Port Elizabeth (of 64 excluded Eastern Cape facilities, three had ≥10 transferred children). We defined children to have successful transfer if they had at least one recorded visit or medication pick up at their assigned transfer facility. Data from records at the transfer facility were abstracted from the first and last clinical visits (or medication pick up), including ART regimen, as well as documentation of deaths and subsequent transfers to other health facilities. Data were collected only from the facility where children were transferred from DNH.

Using information from EMR at DNH and paper-based records at the 16 transfer facilities, we identified three groups of children who were LTF: (1) children LTF at DNH (defined as no clinical visit or medication pick-up >6 months), (2) children with unsuccessful transfer (no recorded visit at the assigned transfer facility), and (3) children with successful transfer (no recorded visit for >6 months according to records at the transfer facility). Children LTF from DNH and those LTF after successful transfer were considered LTF on the last visit/medication pick-up date; children with unsuccessful transfer were considered LTF on the date of transfer. Characteristics of children initiating ART at DNH are compared for children who were ever transferred from DNH to those remaining in care at DNH and for children who had successful transfer and those who had unsuccessful transfer, including age, year of ART, CD4 at entry into care (per the DNH database, no dates provided) and last viral load measure (absolute measure) at DNH which we calculated and defined as the last viral load up to one year prior to transfer or last recorded visit at DNH. The lower limit of detection for viral load tests changed over time and by 2015 was <50 copies/mL. We report data on last viral load from the DNH database with <400 copies/mL as the lowest category because most children had this measure taken prior to 2015.

All children LTF who met the following criteria were considered for community tracing: (1) available contact information, (2) LTF after December 2010, and (3) ≤15 years of age as of December 2015. Community health workers and health facility social workers, accompanied by study staff, conducted home visits using the last recorded home address available in health facility records. Caregivers who were located through tracing were consented and interviewed about the child’s current health, enrolment in HIV care, and ART adherence. A verbal autopsy was conducted for children who had died. Children were not interviewed for the study. Caregivers were encouraged to bring children back to care if they were reportedly not receiving routine HIV services.

After completing community tracing, the National Health Laboratory System (NHLS) electronic database from all nine provinces in South Africa was searched using names and dates of birth of children who were transferred from DNH and those who were LTF while in care at DNH. We searched for tests called for as part of routine monitoring for children on ART per South African national guidelines, including CD4 cell count (CD4+), viral load, TB diagnostic tests, full blood counts and chemistries [28]. NHLS data were used to identify any laboratory results for children following LTF, results within 18 months of LTF to identify silent (undocumented) transfers and results from January 1, 2015 to April 29, 2016 to determine current engagement in care. Viral load measures from the NHLS database from 2015 and 2016 are reported as absolute measures (copies/mL) with a lower limit of detection of 50 copies/mL.

### Statistical analysis

We conducted a descriptive analysis aimed at characterizing children started on ART at DNH and documenting outcomes; comparison of groups based on transfer status were performed using chi-square tests for categorical variables and Wilcoxon signed rank tests for continuous variables. For the analysis of laboratory data to examine children with any labs after LTF, we included any lab ≥1 month after the last visit date; to identify silent transfers, we used a window of 1–18 months; and for the analysis of current engagement in care, we included lab results within 2015–2016 (for children LTF in 2015, labs had to be ≥1 month after the last visit date).

## Results

### All children initiating ART at DNH 2004–2015

**Figure 1.**
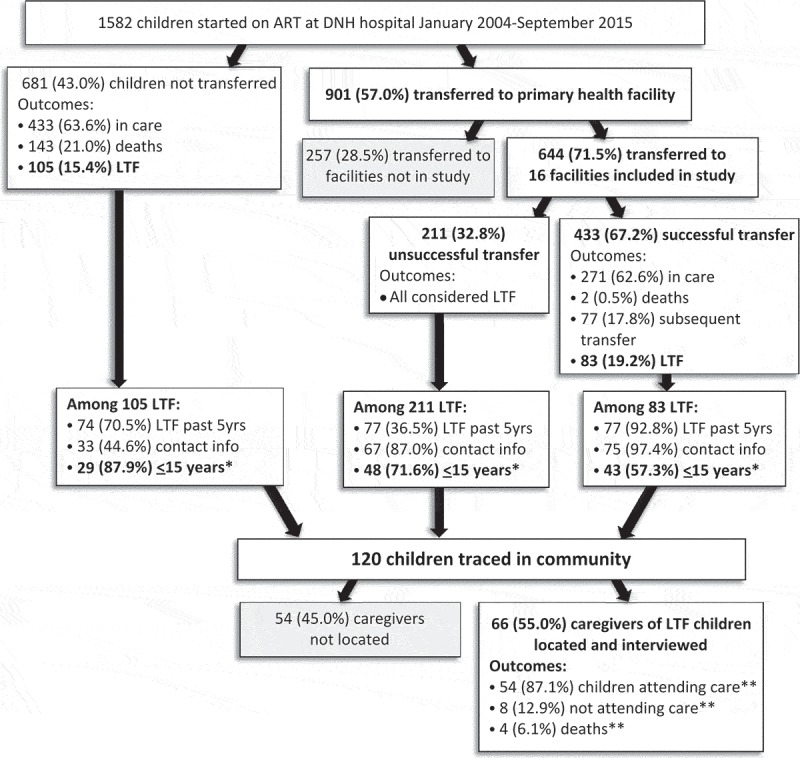
Outcomes of children started on ART at Dora Nginza Hospital in Port Elizabeth, South Africa January 2004–September 2015 (*N* = 1,582). *Children <15 years LTF in last 5 years with contact information. **Among 62 children still alive.

In total, 1,582 children initiated ART at Dora Nginza Hospital (DNH); median age at ART initiation was 4 years [interquartile range (IQR): 1–8] and median CD4+, for those with data (61.4% missing), was 278 cells/mm^3^ [IQR: 119–526] (Table 1). Among all children initiated on ART at DNH, 901 (57.0%) were transferred; median age at transfer was 8 years [IQR: 5–11] and median time from ART initiation to transfer was 37 months [IQR: 21–51]. Compared to 681 (43.0%) children remaining in care at DNH, transferred children started ART in earlier calendar years and were more likely to have initiated stavudine-containing regimens (Table 1). Most children (77.2%) started on treatment at DNH had viral load <400 copies/mL on their last test either before transfer or their last visit (for those who were not transferred) however children who were transferred overall had lower last viral loads compared to children who were not transferred from DNH (Table 1). Among the children who were not transferred, 143 (21.0%) died and 105 (15.4%) were LTF from DNH (Figure 1).

**Table 1.** Characteristics of all children who initiated ART at Dora Nginza Hospital in Port Elizabeth, South Africa by transfer status (*N* = 1,582)

	All	Ever transferred	Never transferred	
	*N* (%)	*N* (%)	*N* (%)	*p*-Value
	1,582 (100.0)	901 (57.0)	681 (43.0)	
**Year of ART initiation**				
2004–2006	381 (24.2)	292 (32.5)	89 (13.1)	<.0001
2007–2009	579 (36.7)	420 (46.7)	159 (23.5)	
2010–2012	416 (26.4)	154 (17.1)	262 (38.6)	
2013–2015	201 (12.8)	33 (3.7)	168 (24.8)	
**Age at ART Initiation, median (IQR)**	4 (1–8)	4 (1–8)	3 (1–8)	0.06
<2	480 (30.3)	229 (25.4)	251 (36.9)	
2–5	488 (30.9)	308 (34.2)	180 (26.4)	<.0001
6–10	490 (31.0)	307 (34.1)	183 (26.9)	
>10	124 (7.8)	57 (6.3)	67 (9.8)	
**Female**	755 (47.7)	425 (47.2)	330 (48.5)	0.64
**CD4 cell/mm^3^ at ART initiation, median (IQR)**	278 (119–526)	276 (131–507)	285 (99–545)	0.32
<200	223 (36.6)	130 (35.0)	93 (39.1)	0.31
200–350	145 (23.8)	98 (26.3)	47 (19.8)	
351–500	83 (13.6)	49 (13.2)	34 (14.3)	
>500	159 (26.1)	95 (25.5)	64 (26.9)	
Missing CD4 at ART initiation	972 (61.4)	529 (58.7)	443 (65.1)	0.01
**ART initiation regimen by age**				
**<3 years**				
ABC+3TC+EFV	10 (1.6)	3 (1.0)	7 (2.2)	<.0001
ABC+3TC+LPV/r	236 (37.4)	65 (20.5)	171 (54.5)	
d4T+3TC+EFV	34 (5.4)	23 (7.3)	11 (3.5)	
d4T+3TC+LPV/r	287 (45.5)	194 (61.2)	93 (29.6)	
Other	64 (10.1)	32 (10.1)	32 (10.2)	
**≥3 years**				
ABC+3TC+EFV	281 (30.1)	86 (15.0)	195 (54.0)	<.0001
ABC+3TC+LPV/r	25 (2.7)	10 (1.7)	15 (4.2)	
d4T+3TC+EFV	465 (49.7)	362 (63.1)	103 (28.5)	
d4T+3TC+LPV/r	22 (2.4)	14 (2.4)	8 (2.2)	
Other	142 (15.2)	102 (17.8)	40 (11.1)	
**Last viral load copies/mL at DNH, median (IQR)**				
>1 million	13 (1.2)	5 (0.9)	8 (1.5)	0.01
10,000–999,999	136 (12.5)	53 (9.4)	83 (15.9)	
1,000–9,999	61 (5.6)	37 (6.6)	24 (4.6)	
400–999	37 (3.4)	17 (3.0)	20 (3.8)	
<400	838 (77.2)	450 (80.1)	388 (74.2)	
Missing viral load	497 (31.4)	339 (37.6)	158 (23.2)	<.0001

*Outcomes after transfer*. Of the 901 children started on ART at DNH who were transferred, 644 (71.5%) were assigned to one of the 16 study facilities (Figure 1) and the majority (84.0%) of transfers occurred in 2010–2012 (Table 2). A total of 433 (67.2%) children had successful transfer and 211 (32.8%) had unsuccessful transfer. Among those with successful transfer, 2 (0.5%) died while in care at the transfer facility, 77 (17.8%) had documented subsequent transfer to another facility and 271 (62.6%) were active in care (Figure 1). Of 433 children with successful transfer, 83 (19.2%) were found to be LTF; median time from transfer to LTF from the transfer facility was 31 months [IQR: 8–45] (Table 2). All 211 (32.7%) children with unsuccessful transfer were considered LTF on the date of transfer from DNH. There were no significant differences in characteristics of children with and without successful transfer (Table 2). Most children who were transferred had viral load <400 copies/mL at the time of transfer with no differences observed between groups.

**Table 2.** Characteristics of children transferred after ART initiation at Dora Nginza Hospital in Port Elizabeth, South Africa to 16 health facilities in the Eastern Cape Province of South Africa (*N* = 644)

	All	Successful transfer	LTF during transfer	
	*N* (%)	*N* (%)	*N* (%)	*p*-Value
	644 (100.0)	433 (67.2)	211 (32.7)	
**Year of ART initiation**				
2004–2006	231 (35.9)	158 (36.6)	73 (34.6)	0.60
2007–2009	314 (48.8)	207 (47.9)	107 (50.7)	
2010–2012	85 (13.2)	60 (13.9)	25 (11.9)	
2013–2015	13 (2.0)	7 (1.6)	6 (2.8)	
**Age at ART Initiation, median (IQR)**	4 (2–8)	4 (2–8)	4 (1–8)	0.14
<2	154 (23.9)	92 (21.3)	62 (29.4)	0.05
2–5	220 (34.2)	153 (35.3)	67 (31.8)	
6–10	229 (35.6)	164 (37.9)	65 (30.8)	
>10	41 (6.4)	24 (5.5)	17 (8.1)	
**Year of transfer**				
2004–2006	1 (0.2)	0 (0)	1 (0.5)	0.55
2007–2009	67 (10.4)	45 (10.4)	22 (10.4)	
2010–2012	541 (84.0)	365 (84.3)	176 (83.4)	
2013–2015	35 (5.4)	23 (5.3)	12 (5.7)	
**Age at transfer, median (IQR)**	8 (5–11)	8 (5–11)	7 (4–11)	0.08
<2	30 (4.7)	18 (4.2)	12 (5.7)	0.05
2–5	185 (28.7)	111 (25.6)	74 (35.1)	
6–10	230 (35.7)	165 (38.1)	65 (30.8)	
>10	199 (30.9)	139 (32.1)	60 (28.4)	
**Median months from ART to transfer (IQR)**	37.3 (20.6–50.6)	38.3 (20.7–51.2)	36.2 (19.3–50.0)	0.41
**Female**	312 (48.5)	210 (48.5)	102 (48.3)	0.97
**CD4 at ART initiation, median (IQR)**	271 (128–494)	276 (120–521)	262.5 (141–434)	0.35
<200	95 (34.6)	67 (35.5)	28 (32.6)	0.31
200–350	76 (27.6)	46 (24.3)	30 (34.9)	
351–500	38 (13.8)	27 (14.3)	11 (12.8)	
>500	66 (24.0)	49 (25.9)	17 (19.8)	
Missing CD4 at ART initiation	369 (57.3)	244 (56.4)	125 (59.2)	0.49
**ART initiation regimen by age**				
**<3 years at ART**				0.97
ABC+3TC+EFV	3 (1.4)	2 (1.5)	1 (1.2)	
ABC+3TC+LPV/r	34 (15.5)	22 (16.7)	12 (13.8)	
d4T+3TC+EFV	18 (8.2)	10 (7.6)	8 (9.2)	
d4T+3TC+LPV/r	141 (64.4)	84 (63.6)	57 (65.5)	
Other	23 (10.5)	14 (10.6)	9 (10.3)	
**≥3 years at ART**				0.68
ABC+3TC+EFV	43 (10.3)	31 (10.5)	12 (9.8)	
ABC+3TC+LPV/r	6 (1.4)	3 (1.0)	3 (2.4)	
d4T+3TC+EFV	280 (66.8)	202 (68.2)	78 (63.4)	
d4T+3TC+LPV/r	13 (3.1)	9 (3.0)	4 (3.3)	
Other	77 (18.4)	51 (17.2)	26 (21.1)	
**Last viral load copies/mL at DNH, median (IQR)**				
>1 million	2 (0.6)	0 (0)	2 (1.8)	0.15
10,000–999,999	25 (7.3)	18 (7.8)	7 (6.3)	
1,000–9,999	26 (7.6)	17 (7.3)	9 (8.1)	
400–999	10 (2.9)	9 (3.9)	1 (0.9)	
<400	280 (81.6)	188 (81.0)	92 (82.9)	
Missing viral load	301 (46.7)	201 (46.4)	100 (47.4)	0.82

### Community tracing

In total, 399 children were identified as LTF: 105 (26.3%) from DNH, 211 (52.9%) at the time of (unsuccessful) transfer and 83 (20.8%) following successful transfer. Most (57.9%) children were LTF in 2010–2012 while 118 (29.6%) were LTF 2013–2015. Community tracing was conducted to reach caregivers of 120 (30.1%) children; 66 (55.0%) were located and interviewed (53 were not located and 1 refused consent). The median age at the time of tracing was 10 years [IQR: 8–12], median age at ART initiation was 2 years [IQR: 1–5] and 71.0% had been LTF for 3 or more years (Table 3). There were no significant differences in the characteristics of children found and not found through community tracing (data not shown). Caregivers reported that 4 (6.1%) children had died.

**Table 3.** Characteristics of children started on ART at Dora Nginza Hospital and subsequently lost to follow-up who were located alive through community tracing (*N* = 62)

	*N* (%)
	62 (100)
**Age at ART Initiation, median (IQR)**	2 (1–5)
<2	23 (37.1)
2–5	25 (40.3)
6–10	13 (21.0)
>10	1 (1.6)
**Age at tracing, median (IQR)**	10 (8–12)
<2	0 (0)
2–5	8 (12.9)
6–10	31 (50.0)
>10	23 (37.1)
**Time since LTF, median (IQR)**	3.5 (1.8–4.8)
<1 year	4 (6.5)
1–2 years	14 (22.6)
≥3 years	44 (71.0)
**Female**	35 (56.5)
**Current caregiver**	
Mother	26 (41.9)
Grandmother	17 (27.4)
Other family/relative	13 (20.9)
Adoptive, foster parent, other	6 (9.7)
**Same caregiver at ART initiation**	50 (80.6)
**Median age of child at HIV diagnosis in months (IQR)**	12 (3–36)
**Child ever admitted to hospital**	42 (68.9)
**Child ever diagnosed with TB, median age at TB diagnosis in years (IQR)**	24 (39.3), 4 (1–7)
**Child told s/he has HIV/AIDS, median age at HIV disclosure (IQR)**	17 (27.4), 11 (10–12)
**Child’s biological mother is alive**	42 (67.7)
**Child’s biological mother is currently taking ARVs**	22 (52.4)
**Home where child is currently living has:**	
Inside tap	50 (75.8)
Electricity	57 (86.4)

**Table 4.** Reasons for removal from care, treatment interruptions and missed visits among children started on ART at Dora Nginza Hospital and subsequently lost to follow-up who were located alive through community tracing (*N* = 62)

	*N* (%)
	62 (100)
**Child not receiving routine HIV care currently**	8 (12.9)
**Reasons child not receiving routine HIV care (*N* = 8)**	
Caregiver does not have time	1 (12.5)
Caregiver does not like going to health facility	1 (12.5)
Caregiver does not want child to take ARVs	1 (12.5)
Child refuses to go	1 (12.5)
Caregiver was away	1 (12.5)
**Caregiver willing to bring the child back to care at a health facility (*N* = 8)**	4 (50.0)
**Child not taking ART**	18 (29.0)
**Reasons child not taking ART (*N* = 18)**	
Child refuses to take ARVs	6 (33.3)
Caregiver does not have time or transport to pick up medication	2 (1.1)
Caregiver does not want child to take ARVs	2 (1.1)
Family disruption	3 (1.7)
Caregiver doesn’t like going to the facility/disclosure issues	3 (1.7)
**Treatment interruption >1 week** (includes 18 children not on ART)	27 (45.8)
**Longest time treatment interruption**	
<1 month	3 (11.1)
1–3 months	5 (18.5)
>3 months	19 (70.4)
**Reasons treatment interruption (*N* = 27)**	
Child was too sick or refused to take	7 (25.9)
Caregiver did not have time or funds	11 (40.7)
Child was not going to health clinic	2 (7.4)
Family disruption	4 (14.8)
Caregiver doesn’t like going to the facility/disclosure issues	2 (7.4)
**Child missed >1 routine care visits since starting ART**	24 (38.7)
**Reasons for missed visits (*N* = 24)**	
No money for transport	5
Child refuses to take ARVs	6 (25.0)
Caregiver does not have time to pick up medication	4 (16.7)
Caregiver does not want child to take ARVs	2 (8.3)
Family disruption	5 (20.8)
Caregiver doesn’t like going to the facility/disclosure issues	3 (12.5)

**Table 5.** Viral load (copies/mL) test results from 2015/2016 for children initiated on ART at Dora Nginza Hospital among those transferred or lost to follow-up (*N* = 749)

	Total (*N* = 749)	LTF from DNH (*N* = 105)	LTF during transfer (*N* = 211)	LTF after successful transfer (*N* = 83)	Not LTF after successful transfer (*N* = 350)
Number (%) of children with VL test results	147 (19.6)	14 (13.3)	36 (17.1)	9 (10.8)	88 (25.1)
Median (IQR)	63 (50–3,715)	5,349 (104–123,266)	50 (50–1,387)	2,072 (220–55,140)	50 (50–486)
>1 million	1 (0.7)	0 (0)	0 (0)	0 (0)	1 (1.1)
10,000–999,999	26 (17.7)	6 (42.9)	4 (11.1)	4 (44.4)	12 (13.6)
1,000–9,999	16 (10.9)	3 (21.4)	5 (13.9)	1 (11.1)	7 (8.0)
51–999	31 (21.1)	2 (14.3)	8 (22.2)	4 (44.4)	17 (19.3)
≤50	73 (49.7)	3 (21.4)	19 (52.8)	0 (0)	51 (58.0)

Overall, 18 (29.0%) of the 62 children were reported to have discontinued ART (Table 4); in addition to the eight children not enrolled in HIV care, an additional 10 children reported to be enrolled in HIV care were also not taking ART. Caregivers cited reasons for discontinuing ART including child’s reluctance, not having time to pick up medication, not liking the facility and family disruptions (housing insecurity, child abandonment and a mother who was incarcerated); no caregiver indicated that clinical care providers had stopped ART. Including the 18 children not currently taking ART, 45.8% reported ART interruptions of longer than 1 week. Most of the periods of treatment interruption were for more than 3 months (70.4%) and resulted from the caregiver not having time or funds to get medications (Table 4).

Most children (68.9%) were reported to have ever had at least one hospital admission and almost 40% had a reported history of tuberculosis. The vast majority of children (87.1%) were reported by caregivers to be currently enrolled in HIV care although receipt of care was not verified at health facilities. The reasons provided by caregivers for why the eight (12.9%) children were not receiving HIV care included the caregiver not having time to take the child, not liking the health facility, not wanting the child to take ART and the child refusing to go (Table 4). Among all children, more than a third (38.7%) were reported to have ever missed a clinical visit (Table 4).

For the four children who died, the ages at death ranged from <12 months to 15 years. Among the deaths, two were children LTF from DNH and two were children LTF during transfer (unsuccessful transfers). Three children died in the hospital and all were reportedly the result of HIV-related illnesses according to caregivers and medical records (shared by two caregivers). All of the children who died were taking ART at the time of death but caregivers reported two children had a history of treatment interruption (data not shown).

### Laboratory monitoring

**Figure 2.**
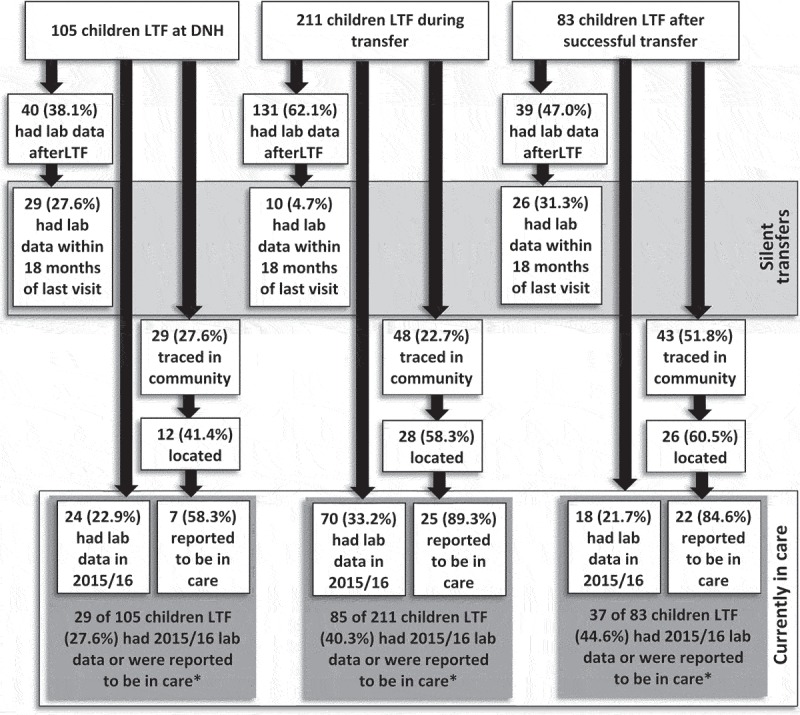
Identifying silent transfer and current engagement in care using laboratory data and community tracing information for 399 children LTF after ART initiation at Dora Nginza Hospital, Port Elizabeth, South Africa. * some children had both lab data and were reported to be in care; the totals for “currently in care” (lower dark grey boxes) are children who had either labs or were reported to be in care. For example, among children LTF at DNH, there were 2 children who had both lab data and were located and reported to be in care, therefore 29/105 or 27.6% overall within this group are reported as “currently in care” (rather than 31).

Using the NHLS database, we found that 210 (52.6%) of the 399 LTF children had lab results indicating that they engaged in care at some point after their date of LTF. The median time from the last known clinic visit to the first lab report after LTF was 27.9 months (IQR: 15.4–36.9). Among 40 (38.1%) children LTF from DNH who had a lab after LTF, the median time was 9.6 months (IQR: 3.8–18.5); 131 (62.1%) children with unsuccessful transfer had lab results after LTF at a median time of 31.8 months (IQR: 27.2–42.0); and 39 (47.0%) children with successful transfer and subsequent LTF had results at median time of 13.6 months (IQR: 5.3–24.4) (Figure 2). Overall, 65 (16.3%) of 399 children identified as LTF had a laboratory result within 18 months of their last visit indicating silent transfer to other health facilities. Children LTF subsequent to successful transfer were most likely to have lab results indicating silent transfer (31.3%) while only 10 (4.7%) children with unsuccessful transfer had indications of silent transfer (Figure 2).

In lab data from 2015–2016, we found that 112 (28.1%) of the 399 children LTF had results, indicating that they were currently engaged in care (Figure 2). Combining recent lab data and reports by caregivers from community tracing, 151 (37.8%) children were currently receiving HIV care. For reference, we also searched 2015–2016 lab data for the 271 children with successful transfer who were determined to be currently active in care (based on medical records) and found that 109 (40.2%) had recent lab monitoring. Also, using laboratory data from 2015/2016, we report the most recent viral loads for all children who were transferred from DNH (including those LTF) and children who were LTF from DNH. We found that among 147 (19.6%) children with results in 2015/2016, the median viral load was 63 copies/mL (IQR: 50–3,715) and almost half of children (49.7%) had viral loads <50 copies/mL (Table 5). Children LTF from DNH had the highest median viral load of 5,349 copies/mL (IQR: 104–123,266) and those who had successful transfer and were not LTF had the lowest median viral load of 50 copies/mL (IQR: 50–486) (Table 5).

## Conclusion

We examined outcomes for children started on ART at a tertiary hospital in Eastern Cape, South Africa and found that among children transferred to care at primary and community health clinics, only two-thirds had successful transfer. These findings show that when children transfer from one health facility, they may be at great risk of being LTF. We then examined outcomes for all children identified as LTF, including those lost during the transfer process. Through community tracing, we were able to locate just over half of the caregivers targeted and found that while 87% of children were reportedly receiving HIV care, 29% were not currently taking ART. The main reason cited by caregivers for the discontinuation of treatment was that the child refused medication which suggests that caregivers face challenges in adhering to care and supporting children to take treatment. Utilizing national laboratory data, we found that 52% of all children who were LTF had laboratory monitoring after the last recorded clinical visit. While this suggests that many children thought to be LTF may have engaged in care, only 16% had lab results within 18 months of LTF indicating silent transfer and continuity of care. Furthermore, through community tracing, we found that 40% of children had missed more than one routine care visit and 46% had a treatment interruption of a month or longer. Taken together, these data suggest that while many children identified as LTF appear to reengage in services, there may be significant gaps in care and treatment interruptions are common, all of which pose significant health risks to HIV-infected children.

Decentralization of HIV care has been considered a critical component in expanding access and improving paediatric HIV care and treatment outcomes. While limited data suggest that children on ART have equivalent or more favourable outcomes at lower-level health facilities [13,16,17], the process of transferring between facilities may be a time of significant LTF, which has not been well documented in children. Our findings suggest that only 67% of children on ART who were transferred from a tertiary hospital to primary and community health clinics ever received care at their assigned health facilities. These results provide new evidence that many children may be LTF during transfer of care and are evidence that greater efforts are needed to ensure that all children who are transferred remain in care. A recent report from the Western Cape of South Africa estimated that 85% of children on ART had successful transfer from a large hospital to lower level health facilities based on lab data [29]. The higher estimate of successful transfer may be a result of better documentation; in the Western Cape a unique patient identifier was used in patient files and laboratory results whereas we used children’s names and dates of birth to locate medical files and lab results. It is possible that due to spelling errors or name changes, we may have not have ascertained all medical or lab records for children. Successful transfer may be more common in Western Cape as a result of more resources and better health infrastructure. Our findings highlight transfer of care from one facility to another as a critical period when children may be LTF. Greater efforts are needed to ensure that all children have successful transfer which should include more complete recording of contact information, better documentation of transfer outcomes at both facilities and active tracing of children who don’t attend care at transfer facilities. It would also be beneficial to have unique medical record numbers used across all facilities and would improve efforts to identify children who do not have successful transfer.

Through community tracing, we were able to locate the caregivers of 66 children who were identified as LTF in the previous five years, which was just over half of all of the children we attempted to locate. We found that 6% of children had died, almost 13% were not attending HIV care at any health facility, and 29% had stopped ART. There was no clear pattern to the reasons for disengagement from care; however, the most commonly cited cause for treatment discontinuation was refusal by the child to take medications which may in part reflect the limitations of current paediatric ART formulations. Only a small number of studies have traced children LTF after ART initiation. A small study from Botswana of seven caregivers of children LTF found that all reported difficulties attending care, many believed the child was well, and others had concerns about disclosure to the child and fears of stigma and discrimination [30]. In data from a large care and treatment programme in Kenya, among 260 children successfully traced after LTF, almost 5% had died, 24% were reportedly in care at another health facility, and 46% were not in care [31]. Reasons for disengagement from care included fear of reprimand, fear of community disclosure, caregivers needing to work, family commitments, and transport challenges. Along with our data, previous findings show that there are both personal and facility-related barriers to care and treatment adherence. In the study from Botswana, caregivers and children proposed ideas to overcome these barriers including weekend clinic hours, shorter wait times to reduce time out of school, and providing a library for patients [30]. These and other innovative strategies are urgently needed to support continued engagement in care.

This analysis of outcomes of children starting ART at a tertiary hospital over a period of 11 years is unique in describing LTF that occurs during transfer from one health facility to another. While there are increasing reports of LTF among children on ART in many RLS, few previous analyses have considered the LTF that occurs during transfer or attempted to verify status of children after LTF. Our finding on LTF during transfer highlights an important issue for paediatric HIV treatment programmes and raises methodological concerns. LTF analyses typically consider documented “transfer out” as an end point mutually exclusive to LTF and death, which implicitly assumes successful transfer for all patients. From our data and those from Western Cape, 15–33% of children appear to have unsuccessful transfer and many of these children may be LTF. In addition, previous studies comparing outcomes of children remaining at facilities where they initiate ART to outcomes of those transferred to primary health clinics do not appear to include LTF during transfer. As a result, these analyses report outcomes only for transferred patients who made it to into the transfer facility, rather than reporting outcomes for all of the patients transferred out of the facility where they initiated treatment; as a result they may be over estimating retention among transfer patients. In light of these findings, we suggest that whenever possible analyses should report LTF during transfer and include retention estimates for all transferred patients (rather than for the subset with successful transfer). For analyses in which transfer outcomes are not measured, we suggest relaxing the assumption that all transfers are successful and propose that some proportion of patients with documented transfer be considered LTF in order to provide more accurate estimates of LTF.

Our analysis also included last viral load taken at DNH for all children prior to transfer or last visit (for those not transferred). We found that children who were transferred had lower viral loads measures overall, as expected based on the report by healthcare providers that a criteria for transfer was stability on treatment; however it should be noted that 16% of transferred children had viral load >1,000 copies/mL. We also report viral load data for children who were transferred and those LTF, both at the time of transfer and the most recent viral load data available in the national database. We found that among the children with recent viral load measure, overall half had viral load <50 copies/mL, including those who were reported to be LTF at the time of transfer. It’s important to note that only a small subset of children transferred and those LTF had recent viral load measures and those who did are likely to be more compliant with care and therefore more likely to have successful treatment outcomes.

While this study provides important new information about outcomes of children on ART, our analysis has some limitations. Not all facilities where children were transferred were included, for feasibility reasons we selected the facilities where the majority of patients were transferred. It is possible that transfer outcomes may differ across facilities. Another weakness, noted above, was reliance on names to identify records which are subject to errors and as a result we may have underestimated the proportion of children with lab results. It is also possible that children who are engaged in care may not have lab results; when we attempted to validate our approach using lab records to verify engagement in care, we found that only 40% of children active in care according to medical records had recent lab results. It is possible that a higher proportion of children were in care but may not have received routine lab monitoring per South African guidelines. Finally, among children located through community tracing we found that 87% of children were engaged in care and only 6% of children had died; however, we did not attempt to trace all children LTF and we were unable to locate almost half of the children we attempted to find. We do know whether the children not targeted for tracing and those not located had different outcomes from those who were included. We also note that children started on ART at hospital facilities such as Dora Nginza, particularly in more recent years following decentralization of care, are likely to be sicker at treatment initiation and therefore may have higher mortality compared to all children with HIV who start ART making our findings on mortality from this cohort not generalizable.

We believe that our findings highlight an important issue not been previously reported, that of LTF occurring during transfer of care for children on ART. Our results shed light on a neglected issue that needs urgent attention – ensuring that all children who are transferred are retained in care. These findings also show that there continue to be barriers to adherence to care and treatment for children and caregivers which must be addressed in order to ensure successful treatment outcomes for all HIV-infected children.
